# The evolving role of placebo in non-inferiority trials: A scoping review

**DOI:** 10.1177/17407745261430850

**Published:** 2026-04-06

**Authors:** Spencer Cho, Shaheer Nadeem, Valentina Ly, Iman Alhafez, Tim Ramsay, Pil Joo

**Affiliations:** 1 Faculty of Medicine, University of Ottawa, Ottawa, ON, Canada; 2 Health Sciences Library, University of Ottawa, Ottawa, ON, Canada; 3 Department of Family Medicine, University of Ottawa, Ottawa, ON, Canada; 4 Ottawa Hospital Research Institute, Ottawa, ON, Canada

**Keywords:** Non-inferiority, placebo, randomized controlled trial, scoping review

## Abstract

**Background:**

Non-inferiority trials can be used for efficacy endpoints or safety endpoints, with indirect and direct comparison to placebo. A less common application of this design involves using placebo as the proposed intervention to challenge standard practices that lack evidence of efficacy. However, the methodology of using a placebo in a non-inferiority trial is poorly described in the literature. We performed a scoping review to map how placebo is utilized in randomized controlled trials with a non-inferiority design, with particular attention to studies positioning placebo as a proposed alternative to existing, but unproven, interventions.

**Methods:**

We conducted a scoping review of randomized controlled trials using non-inferiority designs with a placebo arm, searching six databases without date or language restrictions. Eligible studies were primary randomized controlled trials with at least one placebo arm evaluated under a non-inferiority hypothesis. Data extraction focused on study characteristics, design elements, rationale for non-inferiority design and margin, and analytical practices.

**Results:**

Of 6897 studies screened, 94 met inclusion criteria. Three primary study types were identified: safety (63%), deprescription (20%), and shorter-course (13%) trials. There has been increased use of deprescription and shorter-course studies since 2017. One-third (35%) hypothesized that placebo was non-inferior to active treatment, predominantly in deprescription and shorter-course trials focused on antibiotic use. Most studies (94%) applied non-inferiority analysis to primary outcomes, yet only 22% provided a rationale for non-inferiority design, and despite 96% prespecifying non-inferiority margin, only 41% justified the margin. While 71% used intention-to-treat analysis, only 53% conducted per-protocol analysis. Graphical representation of non-inferiority margins and confidence intervals was reported in 23% of studies.

**Conclusion:**

Placebo is increasingly used in non-inferiority trials aimed at evaluating the necessity of standard interventions, including safety, deprescription, and shorter-course designs. However, many trials lack critical methodological transparency. Future studies should clearly justify non-inferiority designs and margins, use both intention-to-treat and per-protocol analyses, and adhere to the Consolidated Standards of Reporting Trials reporting guidelines to enhance interpretability and rigor.

## Background

In a randomized controlled trial (RCT) for a condition with an already established effective treatment, a placebo-controlled trial for a newly proposed therapy can be unethical.^1,2^ Patients randomized to placebo could be denied access to proven therapies. In such instances, non-inferiority trials can be employed to assess whether a new therapy is similar in efficacy to the current therapy, which is presumed or has been shown to be superior to placebo. Such non-inferiority trials with efficacy endpoints can indirectly support the new therapy’s superiority to placebo while allowing evaluation of other potential advantages in measured outcomes, such as lower cost or fewer adverse effects.^3^ However, when the existing therapy’s superiority to a placebo is less certain, a trial may incorporate both an active-control (to show non-inferiority) and placebo-control (to show superiority).^4^ On the contrary, non-inferiority trials can be designed primarily for safety endpoints which often involve direct comparison to placebo.^5^ Such trials hypothesize that the new proposed therapy will have no excess harm compared to the comparison.

A less commonly discussed application of non-inferiority designs in RCTs involves cases where the current standard of care has not been proven superior to placebo. In such instances, particularly when concerns have been raised about the potential harm of the usual practice, a confirmatory trial may be warranted to test whether the placebo is non-inferior to the existing practice in efficacy. This approach essentially evaluates whether “doing nothing” results in outcomes no worse than the unproven intervention. Here, the placebo acts as the “new” therapy, deviating from the traditional model of comparing a novel active treatment to standard care or placebo. These “myth-busting” or deprescription-oriented studies are well suited for an RCT using a non-inferiority hypothesis and design.^6,7^ For example, older patients with confusion and positive urinalysis without any other infectious signs or symptoms are often given antibiotics, despite a lack of evidence supporting the superiority of such therapy over placebo.^8^ An RCT hypothesizing that the placebo arm (i.e. no antibiotics) would result in a non-inferior rate of delirium recovery in such a population compared to the antibiotic arm could potentially curb one of the most commonly cited reasons for inappropriate antibiotic use in the older population.^9^

Overall, there is limited discussion in the literature regarding the use of a placebo in trials where the placebo is compared to other therapy with non-inferiority intent. Moreover, methodological details for incorporating placebo in non-inferiority RCTs remain poorly defined. A preliminary search of PubMed, the Cochrane Database of Systematic Reviews, the Open Science Framework, and JBI (formerly known as the Joanna Briggs Institute) Evidence Synthesis revealed no current or underway evidence syntheses on this topic.

The objective of this scoping review was to examine how placebo was used in non-inferiority trials. Of particular interest was whether there had been an increasing trend in using placebo as a newly proposed therapy to challenge existing but unproven standard treatments. Nevertheless, we also aimed to explore other contexts in which placebo was incorporated with a non-inferiority intent. For this reason, all trials employing a placebo with non-inferiority intent were included. As our focus was on methodological aspects rather than any specific medical condition, this review sought to map how this particular methodology is being applied across disciplines. Accordingly, a scoping review was deemed the most appropriate approach.^10^

## Methods

We followed published methodological recommendations to conduct this scoping review. The Preferred Reporting Items for Systematic Reviews and Meta-Analyses (PRISMA) Extension for Scoping Reviews Checklist was used to guide the reporting of this scoping review.^11^

### Eligibility criteria

This scoping review included original RCTs using a non-inferiority design with placebo. Eligible studies were required to have one placebo arm that was analyzed in a non-inferiority design. No restrictions were applied regarding patient demographics, settings, interventions, outcomes, or publication language. We excluded post hoc analyses and trials that did not include a placebo arm compared to an active treatment. Observational studies, such as cohort or case-control studies, were excluded. Any non-primary studies, such as letters, editorials, review articles, and case reports or series, were excluded.

### Search strategy

Using the text words in the titles and abstract, and the subject headings, a research librarian at the University of Ottawa (VL) developed a comprehensive search strategy for MEDLINE (Ovid) relating to the two main concepts of non-inferiority and placebo (Appendix A in the Supplementary Material). A second librarian peer-reviewed the search strategy according to the Peer Review of Electronic Search Strategies (PRESS) guidelines.^12^ The search was translated to the other databases and searched without date or language restrictions on May 14, 2024.

### Information sources

Six electronic databases were searched: MEDLINE (Ovid), Embase (Ovid), Cochrane Central Register of Controlled Trials (Ovid), CINAHL (EBSCOhost), Web of Science (Core Collection), and Scopus (see Supplementary Appendix for full search details).

### Data management

Search results were exported to Covidence (Melbourne, Australia), and duplicates were eliminated using the platform’s duplicate identification feature.^13^ The Covidence platform was used for screening and data extraction.

### Selection process

Initial screening of titles and abstracts was conducted independently by three reviewers (S.C., S.N., and P.J.) using Covidence. To ensure consistency, the first 30 papers were screened by all three reviewers and evaluated for discrepancies across the reviewers. Full-text screening was conducted by two reviewers (S.C. and S.N.) to assess eligibility based on predefined inclusion criteria. Any disagreements that arose between reviewers during either stage of screening were resolved by a third reviewer (P.J.). Reasons for excluding studies at the full-text screening stage were documented and included in the PRISMA flow diagram.

### Data extraction

A predefined screening form was used to determine eligibility during full-text screening. A data extraction form was developed and piloted independently by two reviewers (S.C. and S.N.) on three eligible studies to evaluate inter-rater reliability and comprehensiveness of the data extraction form in capturing relevant data. Subsequent modifications were made through team discussions after comparing pilot test results. Two reviewers extracted each article (S.C. and P.J. or S.N. and P.J.), and any difference was resolved by consensus. Extracted data included study design characteristics, details of the active comparator, and the specific outcome analyzed using non-inferiority methodology. In addition, we recorded whether intention-to-treat (ITT) and/or per-protocol (PP) analyses were used, whether the non-inferiority margin was prespecified and accompanied by a rationale, and the justification for using a non-inferiority design with placebo. We also assessed if studies included figures showing confidence intervals and non-inferiority margin. Finally, we noted study types (e.g. safety, deprescription, shorter course, other) and the authors’ conclusions regarding non-inferiority results.

### Data analysis and presentation

We categorized studies as per the goals of the studies by consensus. Findings were presented in tables and graphs and compared between different types of studies. Given that the study is a scoping review, methodological quality and risk of bias were not evaluated.

## Results

**Figure 1.**
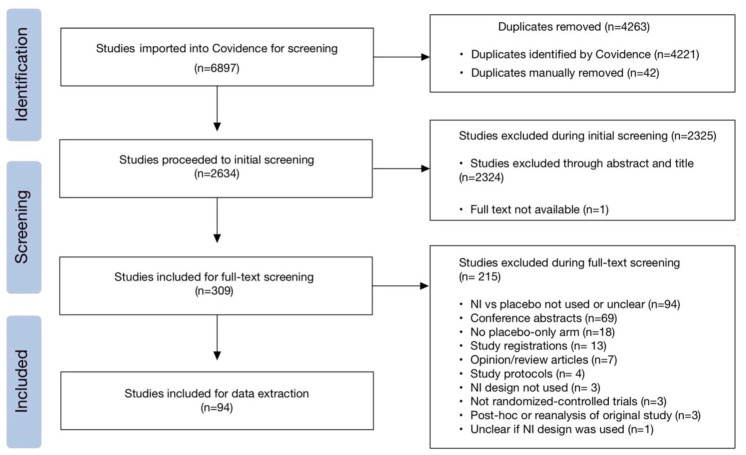
PRISMA flowchart of article selection process.

A total of 6897 studies were imported into Covidence for screening. Of these, 4221 studies were identified as duplicates by Covidence, and an additional 42 duplicates were manually removed. After deduplication, 2634 studies proceeded to initial screening. Following this phase, 309 studies were selected for full-text screening (Figure 1). During the full-text screening process, 215 studies were excluded for reasons such as not using or clearly specifying a non-inferiority placebo design or not being an original report of an RCT. Ultimately, 94 studies met the inclusion criteria and were subjected to data extraction.

We found that largely there were three major types of studies: 59 studies (63%) were safety studies (evaluating whether novel therapies had no excess side effects compared to placebo), 19 studies (20%) were deprescription studies (evaluating whether placebo was non-inferior to active therapy for a specific benefit), 12 studies (13%) were shorter-course studies (where placebo was used to match the omitted portion of a longer regimen), and 4 studies (4%) were classified as other types. In terms of study regions, 25 originated from Europe, 23 studies were from North America, and 21 were multicontinental, accounting for 73% of all included studies. In addition, 3 studies came from Africa, 7 from Asia, 3 from Oceania/Australia, and 12 were from other or unclear regions (Table 1). We note that most multi-continent studies were safety studies.

**Table 1.** Proportion of study locations.

Country	All N = 94	Safety study n = 59	Deprescription study n = 19	Shorter-course study n = 12
Europe	25 (27%)	8 (14%)	6 (32%)	8 (67%)
North America (US/Canada)	23 (24%)	14 (24%)	4 (21%)	4 (33%)
Multi-continent	21 (22%)	20 (34%)	1 (5%)	0
Other/unclear	12 (13%)	10 (17%)	2 (11%)	0
Asia	7 (7%)	4 (7%)	3 (16%)	0
Africa	3 (3%)	1 (2%)	2 (11%)	0
Oceania/Australia	3 (3%)	2 (3%)	1 (5%)	0

US: United States.

**Figure 2.**
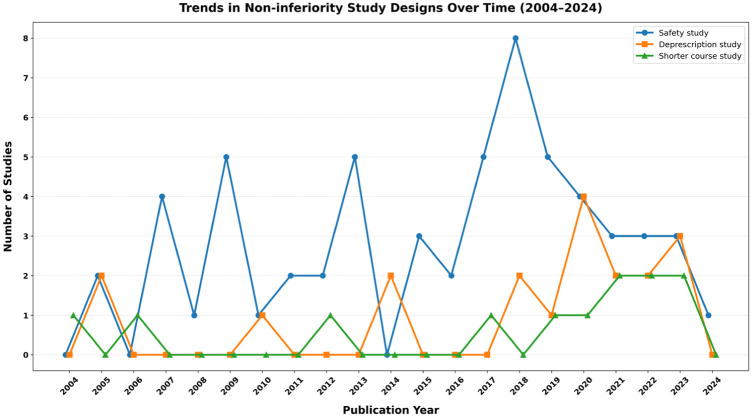
Trends in non-inferiority study designs over time (2004–2024).

Publication years ranged from 2004 to 2024 (Figure 2). Among studies with available data, the median follow-up duration was 3 years, with an interquartile range (IQR) of 1–4 years. We note steady use of deprescription and shorter-course studies starting from year 2017–2018.

Table 2 shows the details of study designs and analyses among selected trials. In 88 studies (94%), the non-inferiority analysis applied to primary outcomes. Only 21 studies (22%) provided an explicit rationale for using a non-inferiority design. A total of 90 studies (96%) prespecified a non-inferiority margin, but only 39 studies (41%) included a rationale for the chosen non-inferiority margin. For analysis methods, 67 studies (71%) reported an ITT analysis, while 50 studies (53%) reported a PP analysis. In terms of hypothesis type, 33 studies (35%) hypothesized that placebo was non-inferior to the active comparison, most of which were deprescription and shorter-course studies. On the contrary, 61 studies (65%) hypothesized that the active comparison was non-inferior to placebo, most of which were safety studies. In addition, 22 studies (23%) included figures showing both confidence intervals and non-inferiority margins. Regarding sample size, 22 studies (23%) were small (≤100 participants), 48 studies (51%) were medium-sized (101–1000 participants), and 24 studies (26%) were large (>1000 participants).

**Table 2.** Study design and analysis, n (%).

	All N = 94	Safety study n = 59	Deprescription study n = 19	Shorter-course study n = 12
NI rationale/margin				
Primary outcome in NI	88 (94%)	54 (92%)	18 (95%)	12 (100%)
Rationale for NI explained	21 (22%)	9 (15%)	7 (37%)	3 (25%)
NI margin prespecified	90 (96%)	57 (97%)	18 (95%)	12 (100%)
Rationale for NI margin offered	39 (41%)	25 (42%)	5 (26%)	7 (58%)
Analysis				
ITT analysis performed	67 (71%)	37 (63%)	16 (84%)	11 (92%)
PP analysis performed	50 (53%)	29 (49%)	9 (47%)	9 (75%)
Hypothesis				
Placebo non-inferior to active therapy	33 (35%)	0	19 (100%)	12 (100%)
Figure showing confidence interval and NI margin	22 (23%)	15 (25%)	4 (21%)	2 (17%)

NI: non-inferiority, ITT: intention-to-treat, PP: per-protocol

Regarding the treated conditions, a wide range of conditions were represented, with the most common being infectious disease (24%) and endocrine (13%) (Table 3). Safety studies examined a wide range of conditions with many studies regarding diabetes (19%). Deprescription studies and shorter-course studies were largely around infectious disease issues (47% and 92%, respectively), essentially all of them examining the need for antibiotic use.

**Table 3.** Proportion of study types across treated conditions organized by system.

Condition	All N = 94	Safety study n = 59	Deprescription study n = 19	Shorter-course study n = 12
Infectious disease	23 (24%)	3 (5%)	9 (47%)	11 (92%)
Endocrine	12 (13%)	12 (20%)	0	0
Healthy/other	9 (10%)	8 (14%)	0	0
Pain/nausea	7 (7%)	4 (7%)	2 (11%)	0
Respiratory	7 (7%)	6 (10%)	1 (5%)	0
Cardiovascular	6 (6%)	4 (6%)	2 (11%)	0
Genitourinary/nephrology	5 (5%)	4 (6%)	1 (5%)	0
Hematology/oncology	5 (5%)	5 (8%)	0	0
Otorhinolaryngology	4 (4%)	3 (5%)	1 (5%)	0
Neurology/psychiatry	4 (4%)	3 (5%)	1 (5%)	0
Reproductive	4 (4%)	2 (3%)	1 (5%)	1 (8%)
Gastrointestinal	3 (3%)	2 (3%)	1 (5%)	0
Surgical	3 (3%)	2 (3%)	0	0
Anesthesia	2 (2%)	1 (2%)	0	0

In terms of primary non-inferiority outcomes, we assessed 63 studies (67%) to show non-inferiority compared to placebo, 19 studies (20%) failed to demonstrate non-inferiority, and 12 studies (12.8%) demonstrated other findings, including superiority.

## Conclusion

### Interpretation

In this scoping review, we explored how placebo has been used within RCTs with non-inferiority design. To our knowledge, our review is the first to map the use of placebo in non-inferiority RCTs across a broader range of trial intents.

Our review process revealed multiple ways that placebo is being used in non-inferiority trials. Many studies involved therapy A versus therapy B versus placebo. In such instances, non-inferiority design often only applied to the comparison between two active treatments (therapy A versus therapy B), while a superiority hypothesis was used against placebo. Such trials were excluded, as placebo was not specifically used with a non-inferiority hypothesis. Among the studies selected for full abstraction that truly compared placebo to another treatment with non-inferiority intention, we found three major types: safety, deprescription, and shorter-course studies. Temporal trends suggest steady use of “safety”-type studies whose hypothesis was often that new active therapy has no excess harm compared to placebo (i.e. active therapy is non-inferior to placebo). These were often for new pharmacotherapeutic agents where such studies were required for approval. For example, the ApoA-I event reducing in ischemic syndromes I (AEGIS-I) trial review by Gibson et al.^14^ included in our review was an industry-funded safety study with the objective of generating safety data to support regulatory approval. On the contrary, “deprescription” and “shorter-course” studies were published sporadically until 2016 but appear to have a more steady presence since 2017–2018. All of these studies hypothesized that the placebo was non-inferior to the current therapy. These deprescription and shorter-course trials predominantly assessed antibiotic use. This approach effectively inverts the traditional framework, positioning placebo as the “new therapy” and (unproven) standard care as the comparator, testing whether less or no intervention is not any worse than the active treatment. This finding may indicate a growing methodological interest in reassessing the necessity of current standard treatments whose effectiveness has not been rigorously assessed in previous trials.

Our review showed that 78% of studies either did not report the rationale for using a non-inferiority design or the rationale was unclear. This finding aligns with observations from a recent scoping review of “Type I discontinuation studies” by Kornder et al.^7^ In this study, 104 trials of both superiority and non-inferiority designs were selected for their goals of evaluating drug discontinuation. The authors found that 58% (60/103) of the studies did not have a clear hypothesis regarding superiority or non-inferiority. Overall, 49% of studies were deemed to have a non-inferiority hypothesis, even though only 10% of the studies expressly stated a non-inferiority hypothesis. This disconnect points to the importance of stating a clear hypothesis and rationale for non-inferiority design. Without a transparent rationale, it becomes difficult to assess the appropriateness of the non-inferiority approach, which may affect the interpretability and credibility of trial findings. The Consolidated Standards of Reporting Trials (CONSORT) extension for non-inferiority and equivalence trials also recommends including the rationale for the use of non-inferiority design.^3^

Our results also speak to the analytical and reporting practices within these non-inferiority trials. While 71% of studies used ITT analysis, only 53% reported PP analysis. This is notable given that both ITT and PP analyses are recommended in non-inferiority trials.^3,15^ The rationale for dual analysis is that ITT may bias toward a non-inferiority conclusion by including non-adherent patients and diluting treatment effects, whereas PP analysis tests the effect under ideal adherence. When findings are consistent across both analyses, the strength of the non-inferiority conclusion is bolstered.^4^ Conversely, discordant results between ITT and PP analyses may cast doubt on the validity of non-inferiority claims. Therefore, the omission of PP analysis in nearly half of the studies in our review raises concerns about risks of misinterpretation or overstated conclusions in favor of non-inferiority.

In addition, less than one quarter of studies included graphical representations that displayed both the non-inferiority margin and confidence intervals. This is an element also recommended by CONSORT extension for non-inferiority trials.^3^ These figures serve an important function by allowing readers to visually assess whether the treatment effect lies within the prespecified non-inferiority margin and to better understand the precision of the estimate. The limited use of such figures, as seen in our review, may reduce readers’ ability to evaluate for themselves the treatment effects and degree of statistical uncertainty, leading to readers having to rely solely on the authors’ conclusions. These concerns about methodological interpretation and reporting are exemplified by a 2021 study by Bennett et al.^16^ The trial concluded non-inferiority of bisoprolol compared to placebo; however, our analysis of the reported results suggests this conclusion may not be statistically justified. The non-inferiority margin in this study reportedly was a 30% drop in area under the curve with bisoprolol versus placebo (or calculated −1.38 in difference). The reported point estimate and confidence interval for the area under the curve between bisoprolol and placebo (−0.41 [−1.67, 2.49]) crossed this threshold. We also note that the confidence interval reported is not symmetrical around the point estimate which may point to a possible typographical error. This discrepancy highlights the potential for divergent interpretations of non-inferiority results between the authors and readers of the study. Notably, the Bennett et al. study reported a non-inferiority margin and the result in a different format (non-inferiority margin in % and result in absolute difference) and did not include a figure displaying the non-inferiority margin alongside confidence intervals. Both factors limit the interpretability of the result to the readers unless further statistical analysis is performed by the readers while making assumptions that could not be verified from the paper itself. Despite reaching out to the authors for clarification, we did not receive a conclusive explanation for the interpretation.

Similarly, the majority of studies did not justify their selected non-inferiority margin. In particular, only one in four deprescription studies offered rationale for non-inferiority margin. The non-inferiority margin is a critical component of trial validity, as it defines the threshold below which a treatment is considered “not meaningfully worse” than its comparator. Arbitrary or poorly justified margins risk accepting clinically inferior treatments or rejecting those that are meaningfully equivalent. Another concern is that the selection of an unjustifiably wide margin can be used to manipulate study outcomes. For example, allowing a trial that would be considered “negative” (i.e. showing a meaningful difference or lack of equivalence) to be interpreted as a “positive” if a more lenient non-inferiority margin is applied. Mauri and D’Agostino emphasize the importance of explicit justification for non-inferiority margins, grounded in both clinical and statistical reasoning, and caution against the use of excessively broad thresholds that can compromise interpretability and validity.^15^ Our findings are consistent with a 2024 systematic review conducted by Sengul et al.,^17^ which examined the methodological quality of non-inferiority trials and found that margin justification, while improving from 36% in 2014 to 57% in 2019, remained suboptimal. Together, these results suggest ongoing variability in the reporting of non-inferiority margin determination and point to the need for clearer reporting standards, particularly regarding clinical reasoning and statistical justification.

### Strengths and limitations

Strengths of this review include a comprehensive search strategy and detailed methodological mapping. The study design and search strategy were developed in collaboration with an academic health sciences librarian at the University of Ottawa and peer-reviewed by a second academic librarian using PRESS guidelines to ensure methodological rigor. We searched six major databases across a broad range of disciplines and imposed no language restrictions, allowing for the inclusion of studies published in non-English languages. In addition, our screening and data extraction processes were independently conducted and pilot tested by multiple reviewers to improve consistency and reduce bias. Despite the strengths of this review, several limitations should be noted. Some relevant studies may have been missed, particularly those in non-indexed journals, despite our efforts to ensure a broad search approach. In addition, this review reflects practices up to the search date (May 14, 2024) and does not capture recently completed trials. Moreover, no gray literature search was completed as part of this scoping review.

### Future research implications

We anticipate more deprescription and shorter-course studies employing placebo and non-inferiority designs will be conducted in the near future. We hope that our review illustrates that such myth-busting trials have been successfully carried out and will encourage well-designed future trials to reduce harm from unnecessary and unproven therapies that are currently accepted as the standard care. In order to increase acceptability of the results, the design and reporting of these studies should ensure that clear rationales for non-inferiority design are offered. Non-inferiority margins should be determined and justified a priori. Investigators should consider both ITT and PP analysis to be included in the analysis. Clear, and preferably graphical, reporting of the results including confidence intervals and the non-inferiority margin would enhance interpretability of the study. Adherence to the CONSORT reporting guideline is recommended.

### Summary

This scoping review maps current practices in trials utilizing placebo with non-inferiority comparison and highlights a relatively novel application of non-inferiority methodology, where placebo is used in place of an active intervention. The number of such studies with the aim of deprescription and shorter-course therapies appears to have increased in recent years. Many studies exhibited methodological shortcomings, including failure to report a clear rationale for using a non-inferiority design or how the non-inferiority margin was determined. These findings point to the evolving role of placebo in non-inferiority trials and highlight the need for improved methodological transparency in future trials.

## Supplemental Material

sj-docx-1-ctj-10.1177_17407745261430850 – Supplemental material for The evolving role of placebo in non-inferiority trials Supplemental material, sj-docx-1-ctj-10.1177_17407745261430850 for The evolving role of placebo in non-inferiority trials by Spencer Cho, Shaheer Nadeem, Valentina Ly, Iman Alhafez, Tim Ramsay and Pil Joo in Clinical Trials
