# Supplementary material for: The evolving role of placebo in non-inferiority trials: A scoping review
Source: Clin Trials. 2026 Apr 6;23(4):439–46. doi: 10.1177/17407745261430850 (PMC13451599; doi:10.1177/17407745261430850)
Supplement: Supplementary file 1 [file sj-docx-1-ctj-10.1177_17407745261430850.docx]

**Appendix A: Search Strategies**

| **Appendix A1. Medline (Ovid) Search Strategy** Ovid MEDLINE(R) ALL <1946 to May 13, 2024> | | |
| --- | --- | --- |
| # | **Searches** | **Results** |
| 1 | equivalence trials as topic/ and (inferior* or noninferior*).ti,ab,kf. | 477 |
| 2 | intention to treat analysis/ and (inferior* or noninferior*).ti,ab,kf. | 228 |
| 3 | equivalence trial.pt. and (inferior* or noninferior*).ti,ab,kf. | 939 |
| 4 | ((non-inferior* or noninferior* or "not inferior*") adj4 (design* or trial* or study or studies or margin or margins or test* or analy*)).ti,ab,kf. | 10936 |
| 5 | (NI adj (design* or trial* or study or studies)).ti,ab,kf. | 82 |
| 6 | or/1-5 | 11148 |
| 7 | placebos/ | 35949 |
| 8 | placebo effect/ | 5228 |
| 9 | (placebo* or sham or shams).ti,ab,kf. | 358289 |
| 10 | or/7-9 | 371538 |
| 11 | 6 and 10 | 946 |

| **Appendix A2. Embase (Ovid) Search Strategy** Embase Classic+Embase <1947 to 2024 May 13> | | |
| --- | --- | --- |
| # | **Searches** | **Results** |
| 1 | "equivalence trial (topic)"/ and (inferior* or noninferior*).ti,ab,kf. | 105 |
| 2 | intention to treat analysis/ and (inferior* or noninferior*).ti,ab,kf. | 1764 |
| 3 | ((non-inferior* or noninferior* or "not inferior*") adj4 (design* or trial* or study or studies or margin or margins or test* or analy*)).ti,ab,kf. | 17335 |
| 4 | (NI adj (design* or trial* or study or studies)).ti,ab,kf. | 173 |
| 5 | or/1-4 | 18035 |
| 6 | placebo/ | 423465 |
| 7 | placebo effect/ | 7951 |
| 8 | sham procedure/ | 16076 |
| 9 | (placebo* or sham or shams).ti,ab,kf. | 531483 |
| 10 | or/6-9 | 696178 |
| 11 | 5 and 10 | 1751 |

| **Appendix A3. Cochrane Central Register of Controlled Trials (Ovid) Search Strategy** EBM Reviews - Cochrane Central Register of Controlled Trials <April 2024> | | |
| --- | --- | --- |
| # | **Searches** | **Results** |
| 1 | equivalence trials as topic/ and (inferior* or noninferior*).ti,ab,kf. | 288 |
| 2 | intention to treat analysis/ and (inferior* or noninferior*).ti,ab,kf. | 208 |
| 3 | ((non-inferior* or noninferior* or "not inferior*") adj4 (design* or trial* or study or studies or margin or margins or test* or analy*)).ti,ab,kf. | 15491 |
| 4 | (NI adj (design* or trial* or study or studies)).ti,ab,kf. | 45 |
| 5 | or/1-4 | 15577 |
| 6 | placebos/ | 27041 |
| 7 | placebo effect/ | 2240 |
| 8 | (placebo* or sham or shams).ti,ab,kf. | 394753 |
| 9 | or/6-8 | 402763 |
| 10 | 5 and 9 | 1213 |

| **Appendix A4. CINAHL (EBSCOhost) Search Strategy** | | |
| --- | --- | --- |
| # | **Searches** | **Results** |
| S11 | S6 AND S10 | 414 |
| S10 | S7 OR S8 OR S9 | 91,525 |
| S9 | TI ( placebo* or sham or shams ) OR AB ( placebo* or sham or shams ) | 87,267 |
| S8 | (MH "Placebo Effect") | 2,479 |
| S7 | (MH "Placebos") | 14,028 |
| S6 | S3 OR S4 OR S5 | 6,205 |
| S5 | TI ( "NI" N1 (design* or trial* or study or studies) ) OR AB ( "NI" N1 (design* or trial* or study or studies) ) | 54 |
| S4 | TI ( (non-inferior* or noninferior* or "not inferior*") N4 (design* or trial* or study or studies or margin or margins or test* or analy*) ) OR AB ( (non-inferior* or noninferior* or "not inferior*") N4 (design* or trial* or study or studies or margin or margins or test* or analy*) ) | 6,164 |
| S3 | S1 AND S2 | 112 |
| S2 | TI ( inferior* or noninferior* ) OR AB ( inferior* or noninferior* ) | 38,152 |
| S1 | (MH "Equivalence Trials") | 138 |

| **Appendix A5. Web of Science (Core Collection) Search Strategy** Social Sciences Citation Index: 1900 to 2024  Arts and Humanities Citation Index: 1975 to 2024  Emerging Sources Citation Index: 2005 to 2024  Science Citation Index Expanded: 1900 to 2024  Conference Proceedings Citation Index - Science: 1990 to 2024  Conference Proceedings Citation Index - Social Sciences: 1990 to 2024 | | |
| --- | --- | --- |
| # | **Searches** | **Results** |
| 1 | TS=((non-inferior* or noninferior* or "not inferior*") NEAR/4 (design* or trial* or study or studies or margin or margins or test* or analy*) ) | 12587 |
| 2 | TS=("NI" NEAR/1 (design* or trial* or study or studies) ) | 5244 |
| 3 | #1 OR #2 | 17758 |
| 4 | TS=(placebo* or sham or shams ) | 421076 |
| 5 | #3 AND #4 | 1188 |

| **Appendix A6. Scopus Search Strategy** | | |
| --- | --- | --- |
| # | **Searches** | **Results** |
| 1 | ( ( TITLE-ABS-KEY ( ( non-inferior* OR noninferior* OR "not inferior*" ) W/4 ( design* OR trial* OR study OR studies OR margin OR margins OR test* OR analy* ) ) ) OR ( TITLE-ABS-KEY ( "NI" W/1 ( design* OR trial* OR study OR studies ) ) ) ) AND ( TITLE-ABS-KEY ( placebo* OR sham OR shams ) ) | 1385 |
